# Interactions of proteins with biogenic iron oxyhydroxides and a new culturing technique to increase biomass yields of neutrophilic, iron-oxidizing bacteria

**DOI:** 10.3389/fmicb.2014.00259

**Published:** 2014-05-30

**Authors:** Roman A. Barco, Katrina J. Edwards

**Affiliations:** Department of Biological Sciences, University of Southern CaliforniaLos Angeles, CA, USA

**Keywords:** iron-oxidation, mariprofundus ferrooxydans, protein extractions, iron oxyhydroxides, chemolithoautotrophic bacteria

## Abstract

Neutrophilic, bacterial iron-oxidation remains one of the least understood energy-generating biological reactions to date. One of the reasons it remains under-studied is because there are inherent problems with working with iron-oxidizing bacteria (FeOB), including low biomass yields and interference from the iron oxides in the samples. In an effort to circumvent the problem of low biomass, a new large batch culturing technique was developed. Protein interactions with biogenic iron oxides were investigated confirming that such interactions are strong. Therefore, a protein extraction method is described to minimize binding of proteins to biogenic iron oxides. The combination of these two methods results in protein yields that are appropriate for activity assays in gels and for proteomic profiling.

## Introduction

Neutrophilic microbial iron oxidation is a biological reaction that has been known since the early 19th century (Kützing, 1833; Pringsheim, 1949). Yet it remains poorly understood when compared with other thoroughly investigated reactions such as sulfur oxidation, nitrogen fixation or iron reduction due to an array of difficulties including low biomass, lack of pure cultures, and interference of iron oxides with sample processing. The majority of the neutrophilic, iron-oxidizing bacteria (FeOB) that have been isolated so far are obligate iron oxidizers; therefore, they cannot grow on media enriched in organic carbon as other groups of bacteria can (i.e., the iron reducing bacteria). These historical difficulties have contributed to the dearth of data on the basic physiology of neutrophilic FeOB, despite important advances in the understanding of acidophilic and phototrophic FeOB.

There are several methods available to grow neutrophilic FeOB, as recently reviewed by Emerson and Floyd (2005). These methods include the use of gradient tubes, gradient plates, solid media, and liquid media. From these methods, the most useful in isolating novel organisms from the environment has been the gradient tube method. This method consists of using small glass tubes that have a FeS plug at the bottom and a defined freshwater or marine medium stabilized with agarose overlaying the plug. Since the bottom FeS plug diffuses Fe(II) upwards and the O_2_ (g) diffuses downwards into the bottom of the tube, the two opposing gradients meet forming a microaerobic region where FeOB can grow and produce a sharp band of growth. The reddish band of growth containing oxidized iron and cells can then be carefully removed, serially diluted, and subcultured with the whole process repeated numerous times to obtain a pure FeOB culture. This method has been crucial in the isolation of many FeOB including *Sideroxydans* ES-1, *Gallionella* ES-2, *Mariprofundus ferrooxydans*, and recently *Ferriphaselus amnicola* (Emerson and Moyer, 1997, 2002; Kato et al., 2013). But one of the problems is with this method is that it does not yield enough biomass for protein research, with total number of cells ranging 10^6^–10^7^ per tube (Emerson and Moyer, 2002). A minimum of 10^9^ cells would be required to obtain approximately 100–150 μ g of protein (Bremer and Dennis, 1996), which is enough protein yield for proteomic analysis. However, if activity assays (i.e., in-gel or based on spectroscopy) are planned, a minimum of 10^10^ cells should be planned. This would indicate that if the gradient tube method is employed, there would have to be thousands of tubes inoculated per experiment; this is clearly an impractical approach. Alternatively, the gradient plate method (i.e., with and without agarose) provides for more volume (15 mLs) and larger total cell numbers, but the number of plates needed per experiment would still be in the hundreds. Solid media is often not an option to grow autotrophic FeOB, since they do not form classical bacterial colonies. The liquid medium method presented in Emerson and Floyd (2005) offers more volume than the gradient tube and gradient plate methods, but it is still relatively low in volume (40 mLs). An attractive factor in using the liquid medium is that it is more realistic to the true environment than the semi-solid agarose medium, a factor that is important if we want to study the physiology of FeOB in a state that is comparable to their natural environments.

In addition to the above challenges, there is an inherent problem to studying FeOB, which is the presence of iron oxyhydroxides (i.e., both biogenic and non-biogenic) in samples. This problem is known for the extraction of DNA in soil and rock samples, for example, where special commercial soil kits are recommended for consistent and appropriate yields of DNA for Polymerase Chain Reaction (PCR) experiments (Wang and Edwards, 2009). In the case of protein analysis, such amplification is not possible. However, there have been several studies that have shown that consistent protein extraction from sediment, soil, rock and wastewater can be attainable (Ogunseitan, 1993; Benndorf et al., 2007, 2009; Keiblinger et al., 2012). Nonetheless, protein extraction remains the biggest bottleneck in environmental—or laboratory based—proteomics due to the technical challenges ranging mainly from low yields, complexity of matrix, and humic acid interference (Bastida et al., 2009; Keller and Hettich, 2009; VerBerkmoes et al., 2009).

In this manuscript, a way to scale up culturing of the FeOB *Mariprofundus ferrooxydans* in liquid medium is presented for purposes of obtaining high-yields of biomass for subsequent proteomic experiments. We also present a method to separate cells from the stalks of biogenic iron oxides that can be useful for analysis of isolated cells. Both of these methods were shared and shortly described in Saini and Chan (2013) but we expand on the details and present new findings. Strong interactions of proteins with both synthesized and biogenic iron oxyhydroxide are also shown and proven to affect protein extractions. A method to circumvent this problem is presented.

## Materials and methods

### Large batch cultures

To generate enough biomass needed for proteomic analysis, a new large-scale culturing method for *M. ferrooxydans* is described. *M. ferrooxydans*, strain PV-1 (hereinafter PV-1), is grown from a stock culture provided by David Emerson (Bigelow Laboratory for Ocean Sciences, East Boothbay, ME, USA). The liquid medium is adopted from Emerson and Floyd (2005) with modifications appropriate for scaling up to 100 and 800 mL cultures. The large batch cultures are grown in 1 L autoclavable polycarbonate bottles (VWR, Visalia, CA) each with 784 mL of medium. Immediately after autoclaving, the medium is sparged with N_2_ (g) for 30 min. The bottle is then sealed with an autoclaved rubber stopper #6 coated in a thin film of silica gel and capped with an open-top cap. After the bottles cool to room temperature, 15.2 mL of sterile sodium bicarbonate solution and filter-sterilized CO_2_ (g) are added to adjust pH to 6.2–6.5 (1.5 min at 15 psi; must check empirically). This is followed by addition of 800 μ L of vitamin cocktail (ATCC, Manassas, VA), 32 mL of filter-sterilized air and 3.2 mL of filter-sterilized 100 mM FeCl_2_ solution. The culture vessel is inoculated with 40 mL of log-phase PV-1 (approximately 10^7^ cells grown in 100 mL batch culture) and incubated in the dark, horizontally at room temperature without agitation. Iron is added every 24 h. The headspace is gas-exchanged every 24 h with filter-sterilized N_2_:CO_2_ (70:30 v/v) (g) and 32 mL of filtered-air. Active cultures in small bottles are maintained in log-phase by subculturing every 48 h until the start of the experiments. Cell counts to determine cell density for these experiments were performed as described in Emerson and Moyer (2002). Briefly, duplicate samples were fixed with paraformaldehyde solution (0.8% final concentration), stored at 4°C for 1 h and then frozen at -20°C until counted. Slides printed with 4 mm diameter circles (Electron Microscopy Sciences, Fort Washington, PA) were coated with 1% agarose solution and allowed to cool. A sample volume of 4— 2 μ L of resuspended culture sample mixed with 2 μ L of 1 mM propidium iodide solution (Life Technologies, Grand Island, NY)—was loaded within the boundaries of the circle and allowed to dry in the dark. Fifty fields per circle were counted at 100X magnification using an epifluorescent Axiostar Plus microscope equipped with an HBO 50 mercury lamp and Cy3 filter for green light excitation. Alternatively, samples were imaged on a TCS SPE confocal microscope (Leica Mycrosystems, Buffalo Grove, IL) using a 488 nm solid-state laser for excitation and emission wavelengths of 495–550 nm for SYTO 9 dye.

### Protein interaction with iron oxides

For each interaction experiment, the mats of log-phase PV-1 were directly harvested from 2 small batch cultures by pipetting and centrifuged at 10,000 × *g* for 5 min. The pelleted mats were washed with 50 volumes of milli-Q water to 1 volume of mats 3 times. Each washing step was followed by centrifugation at 10,000 × *g* for 5 min. The mats were then resuspended in either 50 mL of 20 mM sodium acetate buffer, pH 5.25 (for experiments with cytochrome *c*), or 20 mM Tris Base buffer, pH 8.0 (for experiments with bovine serum albumin, BSA) and loaded into sealed Econo-Pac® chromatography columns (1.5 × 12 cm) (Bio-Rad, Hercules, CA) in a minimal volume of buffer. The final volume of packed mats of PV-1 was 0.8 mL. The mats were then spiked with 50 μ g of either cytochrome *c* from horse's heart (1 mL of 50 μ g/mL fresh stock in 20 mM sodium acetate buffer, pH 5.25) (Sigma-Aldrich, St. Louis, MO) or BSA (1 mL of 50 μ g/mL fresh stock in 20 mM Tris base buffer, pH 8.0) (Bio-Rad). The sample was allowed to incubate for 15 min at room temperature with resuspension every 5 min. After this incubation period, 5 mL fractions are collected while sodium acetate buffer (for cytochrome *c* experiment) or Tris buffer (for BSA experiment) is continually added to the column. Starting with the 5th fraction, either 0.1 N NaOH solution (for both cytochrome *c* or BSA) or 1 M NaCl/20 mM sodium acetate buffer, pH 5.25 (for cytochrome *c* experiment) or 1 M NaCl/20 mM Tris base buffer, pH 8.0 (for BSA experiment) was added to the column. A total of 14 fractions were collected per experiment, which are performed in duplicates. Protein concentrations were measured by the Bradford Assay (Bio-Rad).

### Preparation of 2-line ferrihydrite

The method of Schwertmann and Cornell (2000) was used to produce 2-line ferrihydrite (2LF). Forty g of Fe(NO_3_)_3_·9H_2_O was dissolved in 500 mL of milli-Q water. To this solution, 330 mL of 1M KOH was slowly added to bring the pH to a value between 7 and 8. The mixture was then aliquoted into 50 mL tubes and washed three times in milli-Q water by centrifuging at 10,000 × *g* for 10 min at 4°C. The pellets were frozen at −20°C for 1 day and oven dried for another day at 50°C. This freezing/drying procedure was repeated 3 times. The dried product was ground to particle size >180 μm and <355 μm. Before the start of the experiments, 1 gram of synthetic 2LF was washed 5 additional times with 50 mL of milli-Q water. The synthetic 2LF was then resuspended in 50 mL of buffer (20 mM sodium acetate, pH 5.25 for cytochrome *c* experiments or 20 mM Tris Base, pH 8.0 for BSA experiments) and loaded into a sealed Econo-Pac® chromatography column in a minimal volume of buffer to cover it. The synthetic 2LF had a volume of 0.8 cm^3^ in the column. Samples were spiked as indicated in the last section and were incubated for 15 min at room temperature with resuspension of materials every 5 min. The mineral was characterized at the Los Angeles National History Museum by X-ray diffraction (XRD) to confirm its identity. Data were recorded using a R-Axis Rapid II (Rigaku, The Woodlands, TX) curved imaging plate microdiffractometer with monochromatized Mo*K*α radiation. Observed *d* spacings and intensities were derived by profile fitting using JADE 2010 software (Materials Data Inc., Livermore, CA).

### Protein extraction

#### Osmotic-shock fraction

A total of 8 L of late-log phase PV-1 culture was harvested by filtration on 0.22 μm mesh black Whatman-Nucleopore polycarbonate filters (GE Healthcare Life Sciences, Piscataway, NJ) with 10 μm support TCTP filter (EMD Millipore, Billerica, MA). PV-1 mats were treated for osmotic-shock by immersing the filters in 30 mL of 40 mM Tris-Base pH 8.5/20% sucrose solution followed by addition of 60 μ L of 0.5 M EDTA, pH 8.0 and stirring slowly at room temperature for 10 min (Neu and Heppel, 1965). The mixture was centrifuged at 14,000 × *g* for 10 min at 4°C and the supernatant discarded. The pellet was resuspended in 30 mL of ice cold, sterile 5 mM MgCl_2_ and incubated on ice for 10 min with slow stirring for osmotic shock (Ausubel et al., 1989). The mixture was centrifuged again at 14,000 × *g* for 10 min. The supernatant was saved and stored at −20°C. The osmotic-shock fraction was quantitated by using the Quick Start™ Bradford Assay (Bio-Rad), according to manufacturer's instructions using an UV-1601 spectrophotometer (Shimadzu Scientific Instruments, Carlsbad, CA).

#### Crude extract

A total of 8 L of late-log phase PV-1 culture was harvested as described above. The filters were incubated in 40 mL of 0.1 N NaOH solution on ice for 15 min with vigorous vortexing for 30 s every 3 min to lyse cells and release proteins. The mixture was centrifuged at 14,000 × *g* for 10 min at 4°C to pellet cellular debris and iron oxides. The clarified supernatant was saved and stored at −20°C. The resulting pellet was immersed in another 40 mL of 0.1 N NaOH solution and processed as described above to maximize protein yields. The combined, clarified supernatants were concentrated and buffer-exchanged with 40 mM Tris-Base, pH 8.5 by using Macrosep 3K concentrators (Pall, Ann Arbor, MI) with a 3 kDa molecular weight cutoff (MWCO) membrane according to manufacturer's instructions. The above procedure resulted in a concentrated orange crude extract containing both insoluble and soluble proteins, which was then frozen at -20°C until further analysis.

### Cell separation

A total of 2.4 L (0.8 L triplicates) of log phase cultures were harvested as described above. The filters were immersed in total volume of 250 mL of cold, filter-sterilized 0.2 M oxalic acid, pH 3.0 for two hours with mixing on a shaker table. The oxalic acid partially dissolves the stalks of iron oxides so that the cells are released. The mixture was centrifuged at 14,000 × *g* for 10 min at 4°C to pellet the resulting white paste material and the cells. The supernatant was discarded and the pellet is resuspended in 8 mL of sterile artificial seawater medium (ASW). The resuspended material (4 mL × 2 tubes) was mixed with filter-sterilized 0.001% resazurin (0.2 mL) and carefully layered over a filter-sterilized Nycodez® solution with 1.3 g/mL density (12 mL) (Axis-Shield, Oslo, Norway) in a sterile, polypropylene 50 mL conical tube. Resazurin does not readily mix with Nycodenz and helps visualize the top layer containing the cells. The tube was centrifuged at 10,000 rpm for 50 min at 4°C to separate cells from the white paste/stalks. Being careful not to disturb the two layers, the top pink layer and some of the Nycodenz were transferred into a new tube. The pink solution was diluted 1:2 with ASW to dilute any remaining Nycodenz and centrifuged at 14,000 × *g* for 10 min at 4°C. The resulting pellet, containing cells, was resuspended in a minimal amount of ASW or buffer of choice and processed for cell counting and/or protein extraction.

### Sodium dodecyl sulfate - polyacrylamide gel electrophoresis (SDS-PAGE)

Proteins were separated on gels at 90 V according to standard protocols (Laemmli, 1970). Soluble (*n* = 2) and solubilized membrane fractions (*n* = 2) were run on 12% TGX™ polyacrylamide gels (Bio-Rad) under reduced and non-reduced conditions (i.e., no dithiothreitol (DTT) and no heating). Gels were stained with Bio-Safe Coomassie Stain (Bio-Rad) for 1 h, destained with HPLC-grade water for 2 h and visualized with a Gel Doc™ XR+ imaging system (Bio-Rad) equipped with a white light transilluminator. Images were analyzed with Image Lab™ software (Bio-Rad). Slices were excised as close as possible to the band of interest with a clean sterile scalpel, stored in HPLC-grade water at 4°C and immediately submitted for proteomic analysis.

### Proteomic analysis

Excised protein samples were submitted to the Children's Hospital of Los Angeles - Proteomic Core Facility for trypsin-digestion and LC-MS/MS on a Thermo LTQ-Orbitrap XL mass spectrometer (San Jose, CA) equipped with an Eksigent (Dublin, CA) Nanoliquid Chromatography 1-D plus system. The resulting MS/MS spectra were searched against the generated proteome of PV-1 in the Uniprot database (accession numbers: NZ_AATS01000001-AATS01000032; Singer et al., 2011) using the Proteome Discoverer SEQUEST Daemon search engine. Protein probabilities were assigned by the Protein Prophet algorithm (Nesvizhskii et al., 2003). The criteria for having a protein identified at >95% probability is that there needs to be a minimum of two unique peptides matching to it. Each peptide was established at >95% probability (<5% probability that it is a false positive match to the spectra) by the Scaffold Local FDR algorithm. Datasets were normalized based on the number of assigned spectra (i.e., number of peptides identified). Theoretical isoelectric points and molecular weights were generated by the ExPASy computational tool Compute pI/Mw by using Uniprot Knowledgebase accession numbers corresponding to the reference proteome of PV-1 (Gasteiger et al., 2005). Isoelectric point bias (b) were calculated as defined by Kiraga et al. (2007); *b* = 100 (*N*_basic_ − *N*_acidic_)/(*N*_basic_ + *N*_acidic_), where *N*_acidic_ and *N*_basic_ represent the numbers of acidic and basic proteins, respectively. Statistical analysis was performed by using JMP®, Version 11 (SAS Institute Inc., Cary, NC). The mass spectrometry proteomics data have been deposited to the ProteomeXchange Consortium (Vizcaino et al., 2014) via the PRIDE partner repository with the dataset identifier PXD000937.

### SEM

The sample was fixed in paraformaldehyde solution (0.8% final concentration), incubated at 4°C for 1 h and stored at −20°C afterwards. It was dehydrated sequentially in 25, 50, 75, 90, and 100% molecular grade ethanol with 10 min per dehydration step. Then it was critically-point-dried and gold coated. Images were taken with the scanning electron microscope JSM-7001F-LV (JEOL, Peabody, MA) at the Center for Electron Microscopy and Microanalysis at USC.

## Results

**Figure 1:**
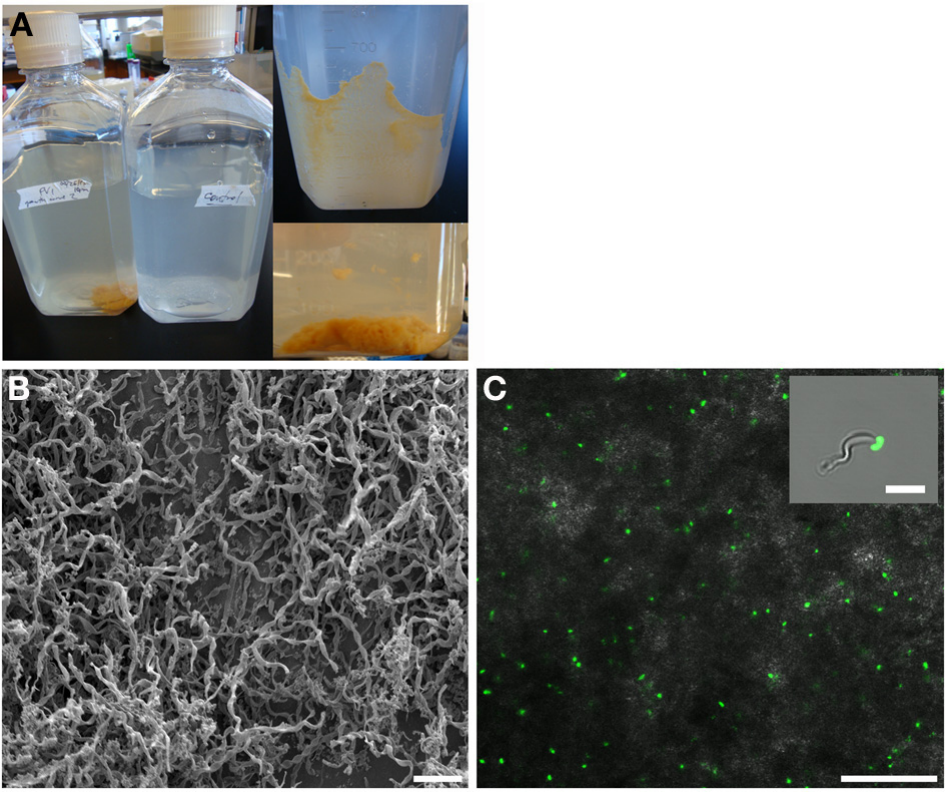
**(A)** Left: large batch culture of *M. ferrooxydans*, strain PV-1 after 1 day of growth, compared to a control. Top-Right: formation of biofilm along the wall of culture bottle after 1 day of growth. Bottom-Right: the biofilm dislodges and forms fluffly flocculent material. **(B)** s.e.m. image of the mats of PV-1 (bar = 10 μm). **(C)** Confocal fluorescent and bright field composite image of the mats of PV-1 (bar = 25 μm). Inset: composite image of isolated PV-1 cell and stalk (bar = 4 μm).

**Figure 2:**
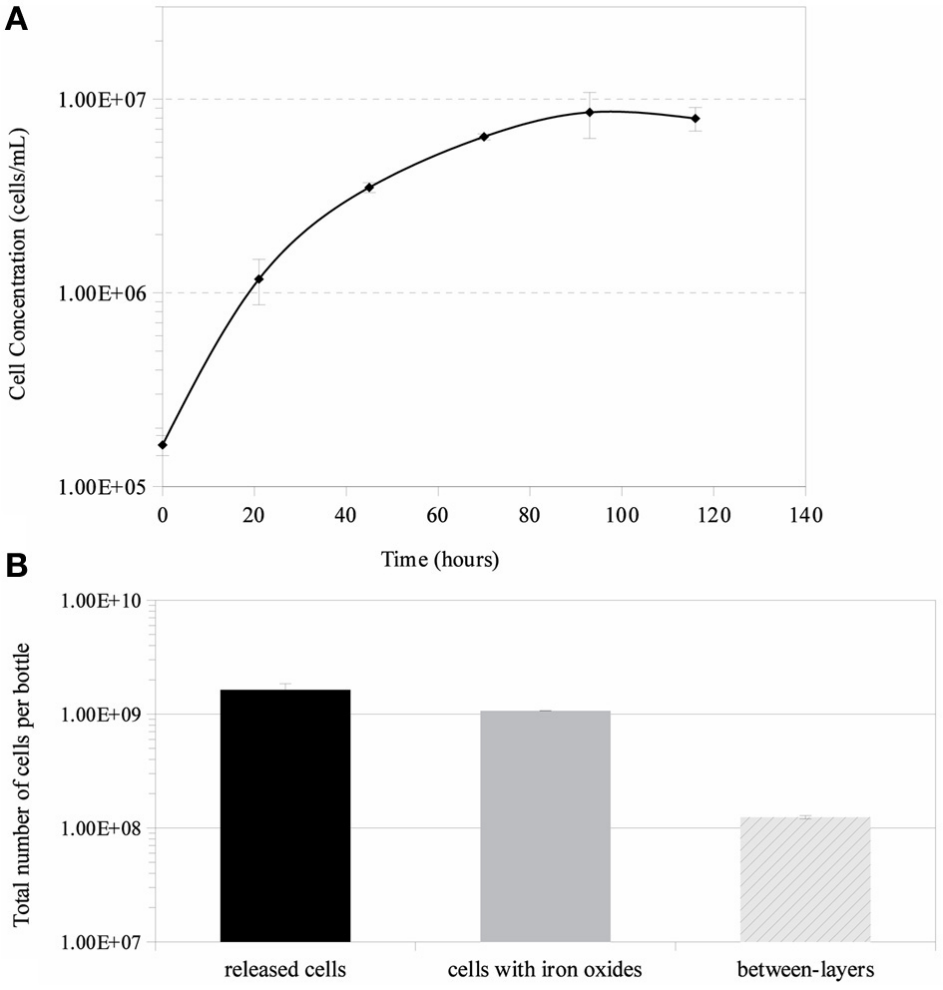
**(A)** Growth curve of *Mariprofundus ferrooxydans*, strain PV-1, using a large-batch culture. Error bars indicate standard deviation from the mean (*n* = 3). **(B)** Overall cell yields obtained by using a method to separate cells from their stalks.

**Figure 3:**
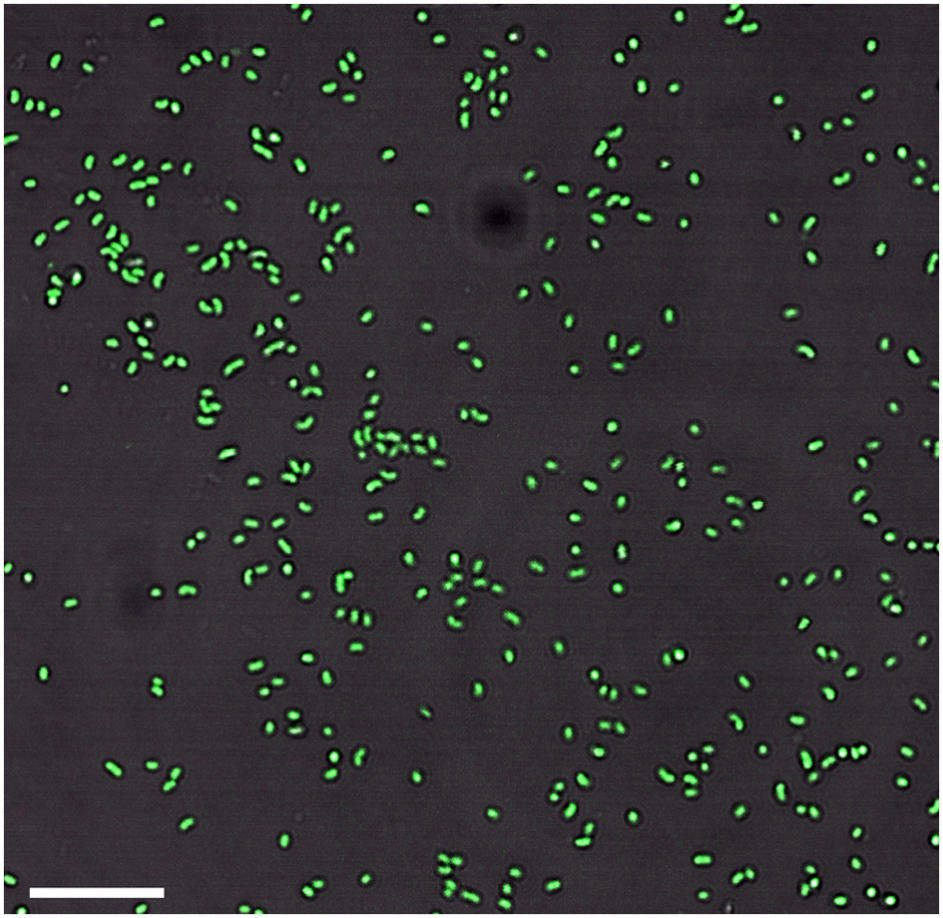
**Enrichment of cells without stalks after the sample goes through the cell-separation method (bar = 10 μm)**.

**Figure 4:**
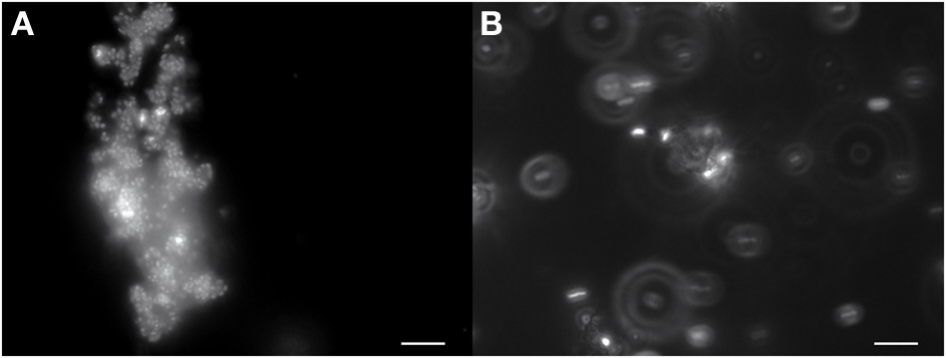
**(A)** PV-1 cells in the mat tend to clump together as the iron in the stalks dissolves with 0.2 M oxalic acid at pH 1. The cells stain red when live/dead stained (dead cells) and stick to a white material that remains undissolved. **(B)** PV-1 cells treated with 0.2 M oxalic acid at a higher pH of 3. The majority of cells are no longer static (hard to focus image) and stain green when live/dead stained (live cells). Some of the white material still remains in the sample and a minority of cells stick to its surface. Bar = 10 μm.

Growth in a large-batch culture was successfully achieved with massive production of biofilm during the first 24 h of culture (Figure 1A). As the biofilm is disrupted, it forms flocculent material that tends to clump together and sink. The biofilm consists of cells and networks of stalks, with mostly biological products of iron oxidation seen as opposed to amorphous products of chemical iron oxidation (Figures 1B,C). Figure 2A shows a representative growth curve of PV-1 growing in a large batch culture. The doubling time is 10 h. The stationary phase is reached at the third day of growth. With an initial concentration of 1.6 × 10^5^ cells/mL, the cell density plateaus at an approximate average of 8.0 × 10^6^ cells/mL. A total yield of 6.4 × 10^9^ cells per single large batch culture is achieved. Figure 2B shows the number of cells that were released from the iron-oxide/stalks matrix following incubation on oxalic acid and centrifugation on a density gradient medium. The number of released cells was approximately 58% of the total number of cells in the large batch bottle (total yield of 2.8 × 10^9^ cells). This fraction was more abundant than the fraction of cells that were still stuck in the iron-oxide/stalks matrix, which corresponded to 38% of the total population. There was a small fraction of cells (<5% of total) that were present in-between the top and bottom layers. Figure 3 shows the enrichment of released cells and the absence of stalks. Incubation with oxalic acid at a pH < 2, dissolves the networks of stalks but makes the cells clump together in a white paste material (Figures 4A,B). Incubation with oxalic acid at pH 3, only partially dissolves the network of stalks so that the cells are released without clumping.

**Figure 5:**
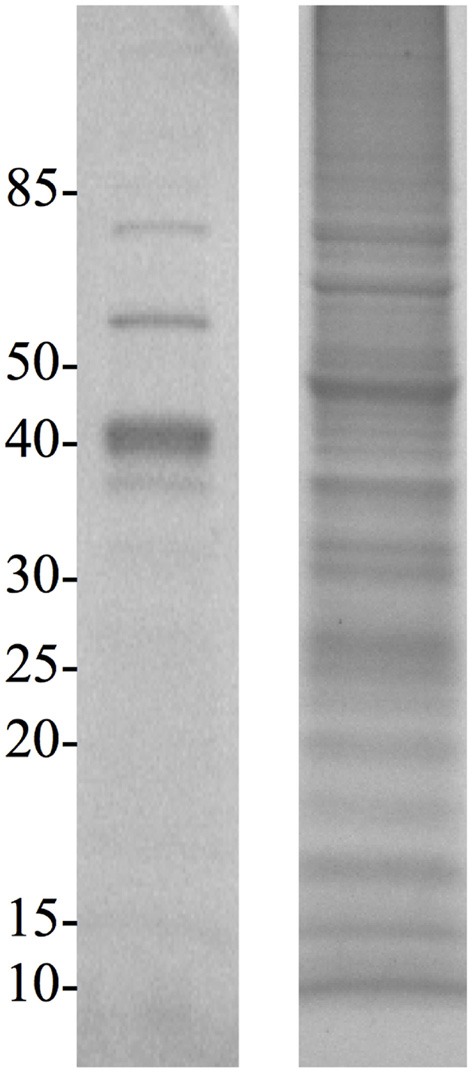
**SDS-PAGE gels of osmotic-shock fraction (left) and crude extract (right) stained with Coomassie Blue**. Molecular masses (in kDa) are indicated on the left.

**Figure 6:**
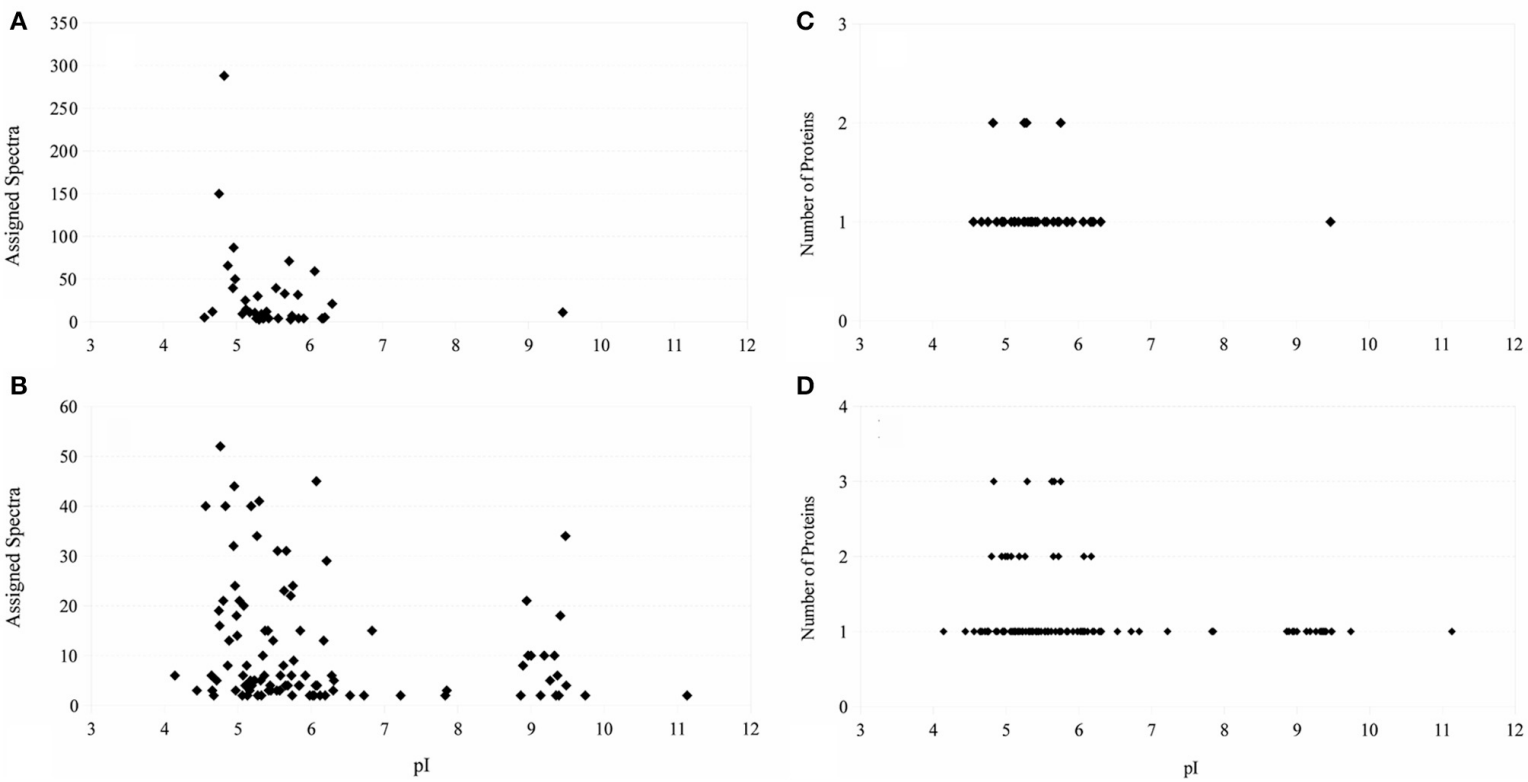
**Left: Number of assigned spectra as a function of isoelectric point of the identified protein (**A**: osmotic-shock sample; **B**: crude extract)**. Right: Number of proteins identified as a function of their isoelectric point (**C**: osmotic-shock sample; **D**: crude extract).

Extraction of proteins following physical disruption of the PV-1 mats (i.e., bead-beating or sonication) yielded either zero bands or only a few faint bands on a coomassie-stained polyacrylamide gel after SDS-PAGE, even when cocktails of various detergents were used (results not shown). Yields were < 1 μ g of protein per L of culture. Similar results were obtained when PV-1 mats were partially dissolved in oxalic acid and the cells were sonicated in the presence of a white material that does not dissolve. Alternatively, a treatment for osmotic-shock that did not disrupt the iron oxides, resulted in more protein extracted (ca. 3 μ g of protein per L of culture) compared to the other disruptive methods including bead-beating, sonication, and acidification. After this sample is run via SDS-PAGE, one of the strongest bands that stains with the coomassie stain was a band around 40 kDa (Figure 5). When a duplicate gel was stained with the heme-stain it showed a weak positive result in an area that matches the molecular mass of 40 kDa. Following these results, a third gel was run via SDS-PAGE and the 40 kDa band was excised and submitted for proteomic analysis. An analysis of the identified proteins indicated that most of them were cytoplasmic; thus, PV-1 cells were lysed by the osmotic-shock treatment (Supplemental Table S1). Remarkably, most of the proteins were acidic or negatively-charged (97.5% of total proteins). Only 1 of 41 identified proteins were basic. In order to determine that the absence of basic proteins was due to a pH effect, the pH of the extraction solution was increased to pH 13 by using 0.1 N NaOH and without physically disrupting the mats. This approach yielded > 50 μ g of protein per L of culture. The sample was run via SDS-PAGE and the 40 kDa band was excised and submitted for proteomic analysis (Figure 5). The results indicate that the protein extraction with NaOH circumvented the bias toward acidic proteins seen in the osmotic-shock treatment (Supplemental Table S1). From 121 proteins identified, 20 were basic (16.5% of total proteins) and with theoretical subcellular localizations including the outer membrane, periplasm, and cytoplasm. Figure 6 shows that more basic and acidic proteins were identified following extraction in 0.1 N NaOH (weighted average pI = 5.83; weighted σ = 1.42), while mainly acidic proteins were identified following the osmotic-shock treatment (weighted average pI = 5.19; weighted σ = 0.61). A non-parametric Kolmogorov Smirnov two-sample test found that the distributions are significantly different (*p* < 0.0001).

**Figure 7:**
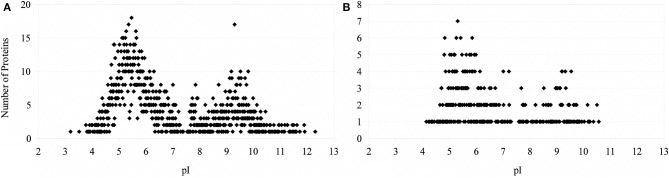
**Number of proteins as a function of isoelectric point. (A)** Theoretical distribution based on the proteome of PV-1. **(B)** Theoretical distribution based on proteins with molecular weights between 33.6 and 51.1 kDa (common range of MW of the proteins identified between osmotic-shock and crude fractions).

**Figure 8:**
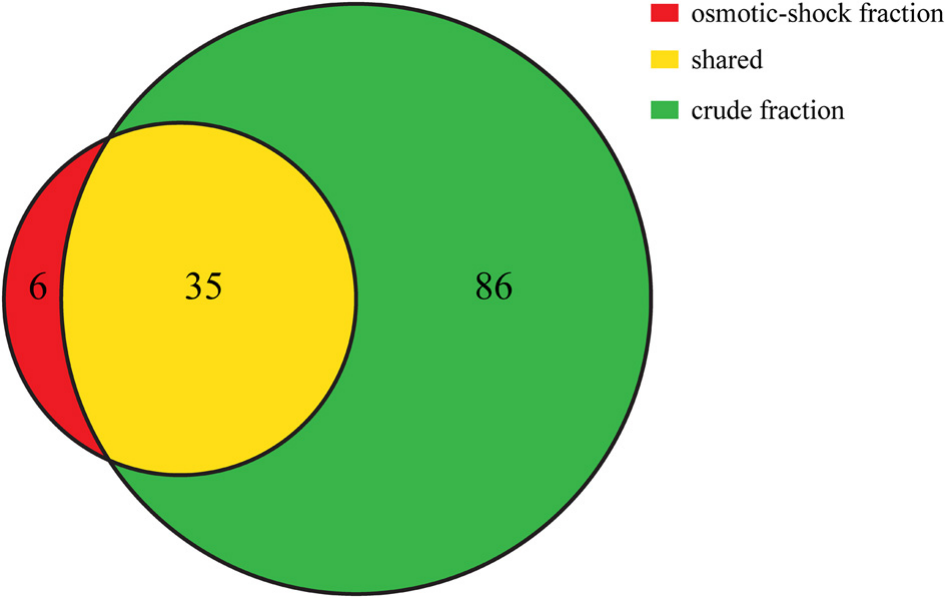
**Venn diagram showing the number of proteins that were uniquely identified in the osmotic-shock fraction (in red) and crude fraction (extracted with NaOH; in green)**. Shared proteins between fractions are shown in yellow. Venn diagram is based on proteins identified with at least 2 peptides. All proteins identified in each fraction are listed in Supplemental Table 1.

The theoretical distribution of the pI values of the proteins generated from the proteome of PV-1 follows a bimodal pattern (Figure 7) that is seen in all organisms so far investigated, including prokaryotes (Schwartz et al., 2001; Knight et al., 2004; Kiraga et al., 2007). A bimodal distribution is also seen if the focus is on a subset of proteins with molecular weights similar to the proteins identified in the bands excised for proteomic analyses. The pI bias of the proteome of PV-1 is acidic (*b* = −36.0), even when only the subset of proteins is taken into account (*b* = −45.0). The pI bias calculated from the proteins extracted with 0.1 N NaOH remains acidic (*b* = −68.6) while the pI bias calculated from the proteins extracted via osmotic shock are extremely acidic (*b* = −95.1) indicating that basic proteins are mostly absent in this latter fraction. Figure 8 shows that the protein extraction with 0.1 N NaOH resulted in more proteins identified, including 85% of the proteins from the osmotic shock fraction.

**Figure 9:**
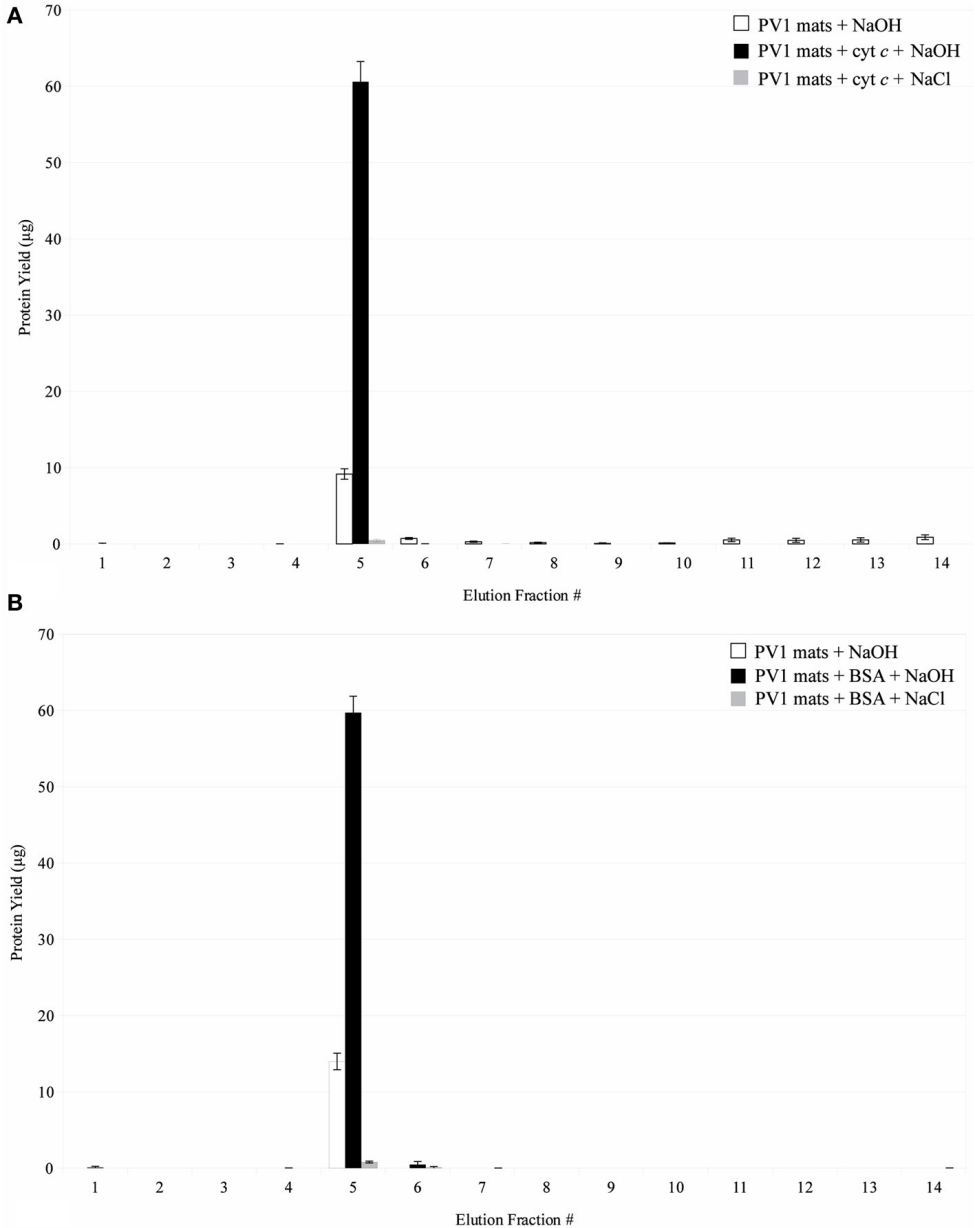
**(A)** Interaction of cytochrome *c* with biogenic iron oxyhydroxide. Fractions 1–4 were eluted in 20 mM sodium acetate buffer, pH 5.25. Fractions 5–14 were eluted in 0.1 N NaOH or 1 M NaCl. Error bars indicate ± one standard deviation from the mean. **(B)** Interaction of BSA with biogenic iron oxyhydroxide. Fractions 1–4 were eluted in 20 mM Tris buffer, pH 8.00. Fractions 5–14 were eluted in 0.1 N NaOH or 1 M NaCl. Error bars indicate ± one standard deviation from the mean.

**Figure 10:**
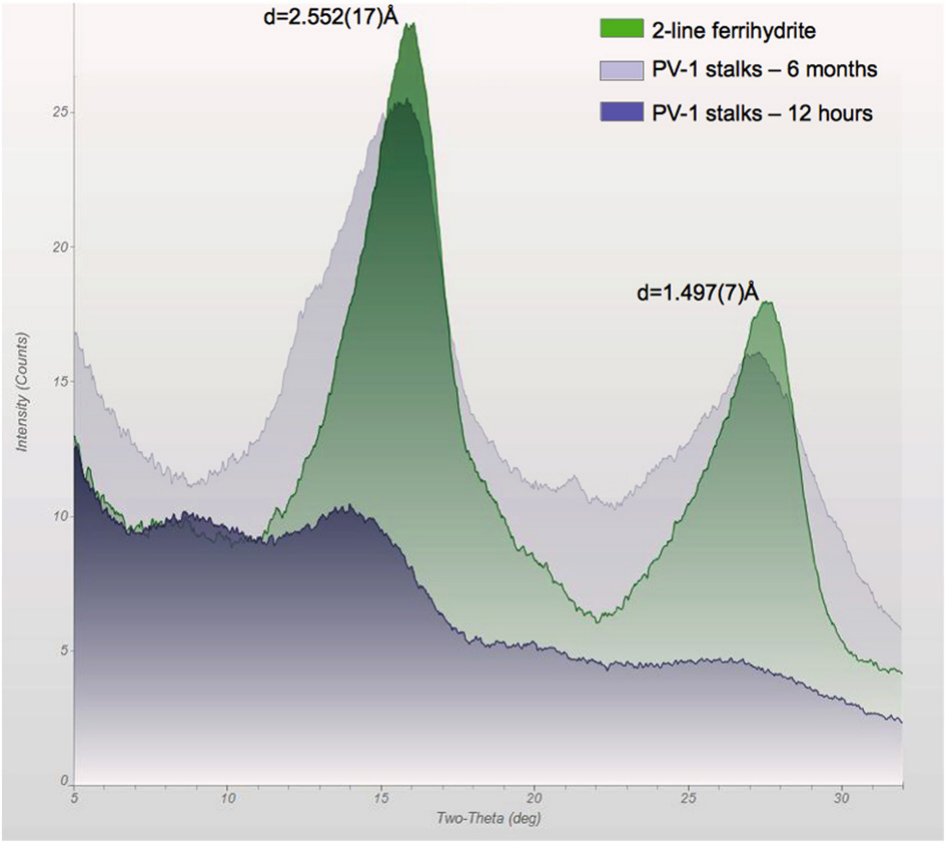
**X-ray diffraction pattern of the stalks of PV-1 compared to synthetic 2LF, showing the development of slightly more crystallized iron oxyhydroxide with time**. At 12 h, the sample was growing at log-phase and the biofilm had an off-white/light yellow color. At 6 months, the sample was reddish. At 3 days of growth, the sample had an orange/reddish color which generated a similar spectrum as the 12 h sample (data not shown). The *d*-spacing values correspond to 2LF for this synthetic material.

Protein interaction experiments were designed to corroborate the results obtained by the proteomic analysis and to test if proteins were actually binding to the PV-1 mats. Figure 9A shows that cytochrome *c*, which is positively-charged at pH 5.25, binds to the PV-1 mats. It remains in the mats even when elution with 1 M NaCl is attempted, suggesting that a force stronger than electrostatic interaction is taking place between the protein and the mats. A pH increase of the elution solution to pH 13 with 0.1N NaOH, eluted effectively the spiked content of cytochrome *c*. At this pH, cytochrome *c* is above its isoelectric point (pI = 10.5); therefore, it would be negatively charged. The point zero charge (PZC) of the stalks of PV-1, the pH at which point the charge is neutral, is not known; however, it is a fair assumption that it will be negatively charged at pH 13. This is because the PZC of pure poorly crystalline 2LF has a value that is 8.11-8.3 (Appelo et al., 2002; Lafferty and Loeppert, 2005), which would indicate that above this pH the surface charge of the mineral is negative. While the composition of the stalk is not known in detail, 2LF is directly associated with it (Edwards et al., 2003; Toner et al., 2009; Chan et al., 2011) (Figure 10). Therefore, at pH 13, negative charge repulsion can explain that cytochrome *c* was eluted at this high pH.

Figure 9B shows that BSA, which is negatively-charged at pH 8.00, also binds to the PV-1 mats. Similarly to cytochrome *c*, it is retained in the mats even when elution with 1 M NaCl is attempted. As mentioned above, this suggests that more direct interactions than electrostatic forces are taking place between the protein and the mats. This can include hydrophobic interactions. A pH increase to pH 13, eluted effectively the spiked content of BSA, which is still negatively-charged because it is above its pI value of 4.75. At pH 8.00; it is unclear what charge the stalks would have because its PZC is not known. At pH 13, because it is such an extreme pH, it is assumed that the stalks would be negatively-charged. Thus, negative charge repulsion can likely explain our observations that BSA was eluted at pH 13.

**Figure 11:**
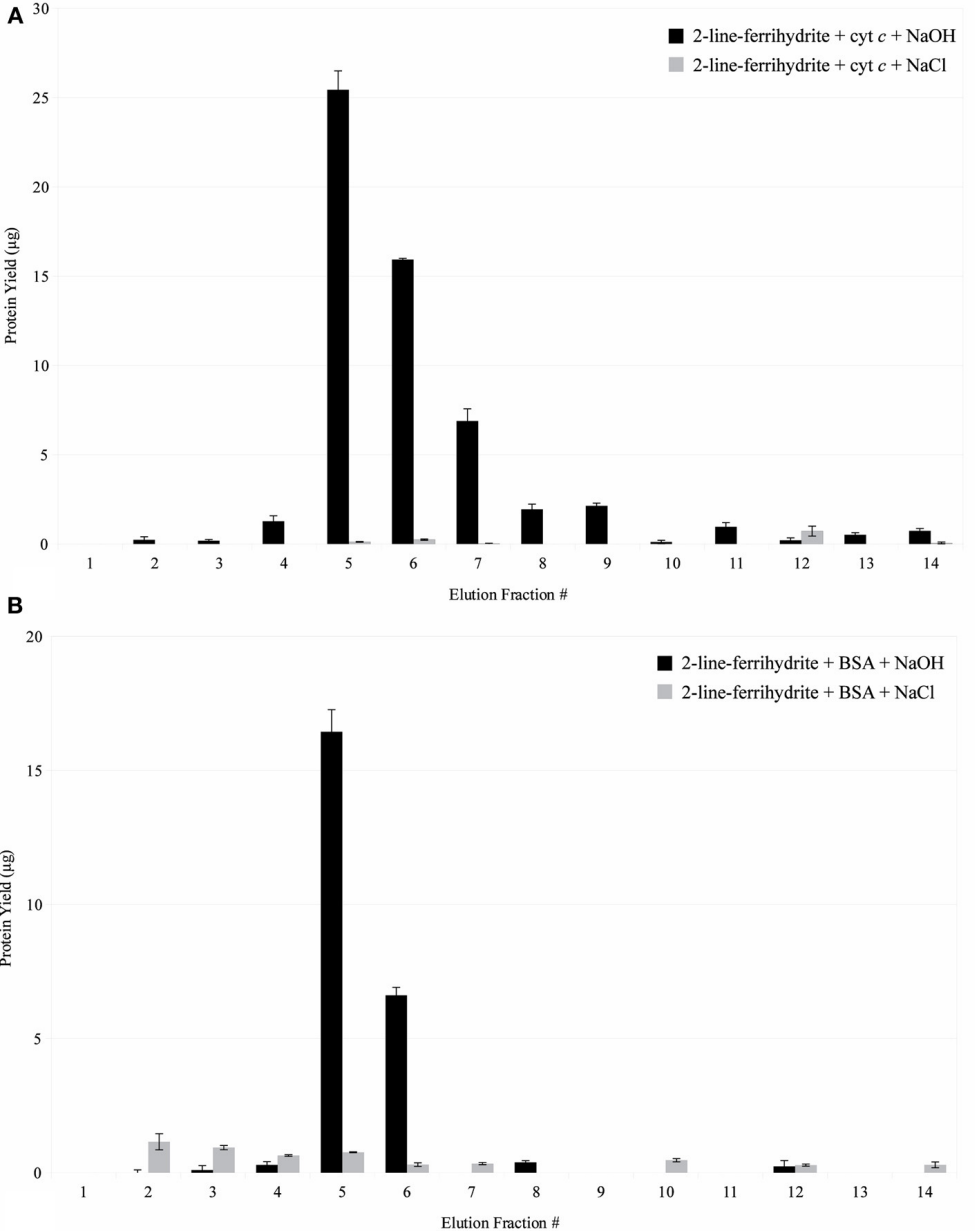
**(A)** Interaction of cytochrome *c* with synthetic iron oxyhydroxide. Fractions 1–4 were eluted in 20 mM sodium acetate buffer, pH 5.25. Fractions 5–14 were eluted in 0.1 N NaOH or 1 M NaCl. Error bars indicate ± one standard deviation from the mean. **(B)** Interaction of BSA with synthetic iron oxyhydroxide. Fractions 1–4 were eluted in 20 mM Tris buffer, pH 8.00. Fractions 5–14 were eluted in 0.1 N NaOH or 1 M NaCl. Error bars indicate ± one standard deviation from the mean.

In comparison to the control using synthetic 2LF (Figure 10), PV-1 mats have a lower retention capacity of the proteins spiked. This indicates that it is easier to desorb proteins from PV-1 mats than from synthetic 2LF. At pH 5.25, positively-charged cytochrome *c* binds to positively-charged 2LF (PZC = 8.11–8.33). In this case, repulsion is expected but the 15 min contact time, allows for the proteins to bind effectively to synthetic 2LF. The desorption of cytochrome *c* from synthetic 2LF was complete but took five fractions of 0.1 N NaOH to achieve it (fractions 5–9). In comparison, cytochrome *c* was desorbed from PV-1 mats effectively with the first fraction of 0.1 N NaOH added (Figure 11A).

Figure 11B shows that at pH 8.00, negatively-charged BSA binds to positively-charged synthetic 2LF. The desorption of BSA from synthetic 2LF is only partially achieved, with synthetic 2LF retaining close to half of the original amount of BSA spiked after elution is attempted with 0.1 N NaOH. In this latter case, the higher protein retention capacity observed in synthetic 2LF can be explained by a combination of electrostatic forces as well as direct mineral-protein interaction (i.e., hydrophobic interaction), which is a type of interaction that can be irreversible (Dobrikova et al., 2007; Rocker et al., 2009; Safi et al., 2011). Spiked BSA was only minimally eluted with 1 M NaCl, which would indicate that the hydrophobic interactions between the protein and mineral play a major role than the electrostatic forces involved.

## Discussion

One of the goals of this study was to develop practical methods to increase biomass of FeOB so that proteomic studies could be realized. Past studies have shown that 10^9^ cells of *Escherichia coli and Streptomyces coelicolor* yield 100–150 μ g and 143–181 μ g of protein respectively (Bremer and Dennis, 1996; Cox, 2004). Using these numbers as guidelines, and the minimum requirements of 30–50 μ g of protein for a full proteomic profile via LC-MS/MS, the goal is to achieve a minimum of 10^9^ *M. ferrooxydans* cells per experiment. This total cell number yields enough protein to use for proteomic analysis, with enough material for a possible technical replicate. However, if spectroscopic analysis or activity in-gel assays are planned (e.g., staining with heme-stain), then a minimum cell number of 10^10^ needs to be targeted so that protein yields are closer to 1 mg. The growth curve obtained from the large batch cultures (Figure 1) show a cell density of > 10^6^ cells/mL and that a total number of cells of 10^9^ can be achieved per bottle within 24 h. This can be compared to total cell numbers of 10^7^ within 24 h using other types of cultivation methods such as the gradient plate method (Emerson et al., 2007). The growth curve also shows that the cell densities have intermediate values compared with other previously published cell densities of 10^6^ to 10^7^ cells/mL (Emerson et al., 2007; Chan et al., 2011). Cultivation with 10 large bottles (i.e., total of 8 L of culture), following the method presented herein, produces enough biomass (10^10^ cells) for proteomic analysis and in-gel activity assays. Alternatively, a more concentrated inoculum should also increase final cell yields.

The doubling time of 10 h reported in this study was calculated for the first 45 h of growth. If it is calculated during the first day of growth, as seen in a recent paper by Kikuchi et al. (2014), the doubling time is 7 h which is the same value obtained with Kikuchi's diffusion chamber method. The longer than usual doubling times of PV-1 that were determined by Kikuchi et al. (2014) when testing the liquid batch culture method may have to do with the addition of a single -high- dose of Fe^2+^ at *t* = 0 h, a practice that is not consistent with the protocols in Emerson and Floyd (2005) for liquid batch cultures which call for daily addition of lower concentrations of Fe. Addition of excessive amounts of Fe at *t* = 0 h sequesters more than usual levels of phosphate in the medium, which in turn increases the doubling time of PV-1. The identification of two proteins that are associated with ABC-phophate-transporters (NCBI GI numbers: 114777113 and 114777114) identified in this study, support the explanation that PV-1 goes through phosphate limitation by the time of harvest (third day of growth). Therefore, even higher levels of Fe in the medium should exacerbate this effect.

The other goal of this study was to present methods to facilitate protein extraction for proteomic analysis of cells that are in close contact with biogenic iron oxides. Experiments on the interactions of proteins with the mats of PV-1 originated with extractions using disruptive forces such as bead-beating, sonication, and even acidification of sample; all of these methods consistently resulted in low yields of proteins in the presence or absence of detergents. Another hint was given when the non-disruptive osmotic-shock method was employed to enrich for cytochrome *c;* instead an enrichment of acidic proteins was obtained. Only one basic protein (pI = 9.4) was identified, which happened to be one of the proteins associated with ABC phosphate-transporters (NCBI GI number: 114777113). By increasing the pH of the extraction solution to pH 13, all the proteins in PV-1's proteome should in theory be above their pI, which would make them negatively charged if they are expressed. This is a different approach from proteomic studies involving acidophilic FeOB where the Fe(III) is already dissolved in a strongly acidic medium or in acidic mine water and sonication of washed cells is not a problem (Ram et al., 2005; Bouchal et al., 2006). However, when this approach is taken for PV-1, a substantial amount of white paste remains, which seems to effectively bind to proteins in solution (i.e., no bands seen in gel stained with coomassie blue). This white paste has been mentioned by Emerson and Moyer (2002) as being organic matter originating from the stalks, but no characterization has yet been made. The use of NaOH along with SDS has long been used in alkaline lysis of bacterial cells (Birnboim and Doly, 1979) and more recently in yeast cells (Kushnirov, 2000). There have been also recent studies on soil proteomics that have used NaOH and SDS as the protein extraction solution, followed by a phenol extraction protocol to remove humic acids (Benndorf et al., 2007). The use of SDS alone can be effective in lysis of marine microorganisms (Fuhrman et al., 1988). In light of the results from these past studies, it is recommended that the NaOH solution used in this study to desorb proteins from biogenic iron oxides be complemented with SDS to maximize cell lysis of neutrophilic FeOB as well as to help solubilize membrane proteins in the crude extract.

The fact that the spiked proteins were easily adsorbed and desorbed from PV-1 mats, and the different behavior in synthetic 2LF, suggests that poorly crystalline iron oxyhydroxide is not the only substrate playing a role in adsorption/desorption of proteins. This can be explained by a close interaction of Fe and an organic polymer, which is consistent with different models that have been proposed for biomineral formation in PV-1 (Chan et al., 2011; Bennett et al., 2014). These results can also suggest that the proteins bound to PV-1 mats have the potential to be more accessible or bio-available than proteins bound to synthetic 2LF.

## Conclusion

A method that yields sufficient biomass for proteomic analysis involving neutrophilic FeOB has been developed. Our method circumvents the problem of protein adsorption onto biominerals and shows that both acidic and basic proteins can be identified. Using the methods herein presented, a proteomic profile of *M. ferrooxydans* was produced but a full analysis will be described elsewhere. Our results indicate that the twisted-stalks produced by *M. ferrooxydans* not only bind strongly to proteins but also can desorb them more easily than synthetic 2LF. This leaves open the possibility that fields of iron mats seen in the ocean floor, such as the recently identified FeMO Deep site at the base of Lōi'hi Seamount, near Hawaii (Edwards et al., 2011), are natural banks of bioavailable adsorbed protein, not only from neutrophilic FeOB, but from the rest of the microbial community surrounding it. This can in turn influence the growth of iron reducing bacteria that are known to be present in iron mats of Lōi'hi Seamount but are hypothesized to be carbon limited (Emerson, 2009).

### Conflict of interest statement

The authors declare that the research was conducted in the absence of any commercial or financial relationships that could be construed as a potential conflict of interest.
